# Pathway Detection from Protein Interaction Networks and Gene Expression Data Using Color-Coding Methods and A∗ Search Algorithms

**DOI:** 10.1100/2012/315797

**Published:** 2012-04-01

**Authors:** Cheng-Yu Yeh, Hsiang-Yuan Yeh, Carlos Roberto Arias, Von-Wun Soo

**Affiliations:** ^1^Department of Computer Science, National Tsing Hua University, Hsinchu 300, Taiwan; ^2^Institute of Information Systems and Applications, National Tsing Hua University, Hsinchu 300, Taiwan

## Abstract

With the large availability of protein interaction networks and microarray data supported, to identify the linear paths that have biological significance in search of a potential pathway is a challenge issue. We proposed a color-coding method based on the characteristics of biological network topology and applied heuristic search to speed up color-coding method. In the experiments, we tested our methods by applying to two datasets: yeast and human prostate cancer networks and gene expression data set. The comparisons of our method with other existing methods on known yeast MAPK pathways in terms of precision and recall show that we can find maximum number of the proteins and perform comparably well. On the other hand, our method is more efficient than previous ones and detects the paths of length 10 within 40 seconds using CPU Intel 1.73GHz and 1GB main memory running under windows operating system.

## 1. Background 

With the large availability of protein-protein interaction data, to identify the corresponding networks such as functional enriched pathways is a big challenge. Protein interaction networks are the assembly of the protein signal cascades that transfer the biological function and information through the pathway, and the proteins in the cell react with the environment by transduction signals. We considered the protein interaction networks from available public databases as a graph in which nodes represent proteins and edges represent protein interactions, and we can apply search method to discover the significant paths which are identified with similar biological processes and functions. Therefore, protein-protein interactions provide important information in assembly of signaling pathways and have great potential for us to understand the cellular mechanism and functions. On the other hand, numerous biological studies used experimental data such as microarray data or GeneChip to identify the expression of the individual gene, and the expression values of genes have the over/underexpressions under some conditions. Integrating both protein interaction network and gene expression data is a popular procedure in bioinformatics research area. Discovering the signaling pathways is to search the linear paths from cell-surface proteins to transcription factor proteins in nucleus. The previous research focused on extracting the specific network structure such as protein complexes from the gene expression data [1]. The definition of the pathway is not the same as uncovering/extracting the linear or tree-type pathways from the protein network. Biologists use the term “linear path” to denote a simple path in the protein interaction network where no node (protein) can occur more than once. Unfortunately, searching a linear path in the protein networks where each node occurs at most once is a NP-hard problem [2]. In the previous study, Steffen et al. applied an exhaustive search to identify the pathways in the protein networks that assumed all the weights of interactions be equal and reliable [3]. This method successfully detected some known pathways in yeast. However, only paths with short length could be detected while the exact search algorithm causes the exponential running time. Recent works estimated the weights of interactions based on high-throughput microarray data, the number of times an interaction referenced in papers or experiments instead of using unweighted interaction in the networks. By assigning scores to protein interactions, Alon et al. proposed a randomized color-coding concept in search of a pathway as a minimum-weight paths searching problem [4]. The color-coding method can reduce the running time comparing with the exhaustive search for longer paths but it still has exponential runtime depending on the number of colors. The basic idea behind color coding is to randomly color each node from 1 and *k* kinds of colors and search for paths with distinct colors instead of searching for paths with distinct nodes. Shlomi et al. formalize pathway detection as pathway query problem that is the problem of finding a path in a labeled and weighted graph that best matches a prespecified query path [5].

Mayrose et al. used color-coding technique to detect the simple paths in a graph for mapping a peptide to the surface graph in protein 3D structure and applied branch-and-bound technique to decrease the search space in the dynamic programming, and it costs about 10-fold speedup of running time [6]. Scott et al. also provides a randomized color-coding and applied dynamic programming method to solve this problem limited to path length above 10 nodes but it requires several hours to execute [7]. They also applied some biological constraints to segment the pathways based on their cellular locations in Gene Ontology database to fit the biological meanings [8]. Hüffner et al. modified the color-coding method by increasing the number of colors and employed a fixed lower bound to find the candidate nodes in interest efficiently [9]. However, there is no way to know the true optimal cost-to-go using dynamic programming, and they still store a lot of space to get the optimal solution. Zhao et al. proposed an integer linear programming (ILP) model to find the signal pathways by utilizing both protein interactions and microarray data [10]. Comparing with previous color-coding methods, this method can recover signal pathways directly by running the program once instead of assembling the candidate pathways. PathFinder used the functional annotation to realize the characteristics of the known pathways and extracted the association rules from the known pathway based on the hierarchical of Gene Ontology (GO) annotation as template. They searched the candidate pathways which contain the characteristic similar to the template and filtered the protein-protein interaction network by integrating other biological knowledge [11]. GO terms include a brief description of the corresponding biological function of the genes, but only 60% of all human genes have associated GO terms, and these terms may be inconsistent due to differences in curators' judgment [12]. Due to the incomplete data, the approaches reduce the probability to extract the pathway.

The previous works show that the color-coding approach is capable of identifying biologically meaningful pathways. The central idea that underlies color-coding is to randomly color each node in the network using a small set of colors and to “hope” that the path becomes colorful. Random colorings need to be tried to ensure that the desired paths are not missed and to find a colorful path can be accomplished in *O*(2^k^ *m*) time for an m-edge graph by dynamic programming which is proved by Huffner's work [9]. The more important way to improve color-coding algorithms efficiently is to derandomize it, and this kind of research was also mentioned in Huffner's paper [9]. However, there is no one focuses on this way to improve the color-coding approach, and that is also the reason we want to do. We presented a new and more efficient method for detecting the simple paths with biological constraints and heuristic function that differs from the above researches. We consider the topology characteristic of biological network such as high-degree nodes and articulation hubs into the color-coding method and applied heuristic method to prune the search space instead of exhaust searching for all the edges in the network. The paper is organized as follows: Section 2 describes the proposed methods in detail.  Section 3 explains the experiments and discusses the results.  Section 4 makes the conclusions. 

## 2. Methods

Our methods consist of four modules: Module 1 integrated the public protein-protein interactions database and assigned weight values of interactions based on the Pearson correlation calculated from microarray data. Module 2 presented specific color-coding techniques based on the significance of biological network topology such as the degree of a node and an articulation hub. Module 3 applied a pruning strategy to speed up the color-coding methods. Module 4 used known pathways to validate the functional enrichment of pathways found by our methods.

### 2.1. Network Construction from Microarray Data and Protein-Protein Interactions Database

The microarray data implies gene expressions information in the biological experiments and characterize protein functions in vitro. The microarray dataset consists of *N* genes and *M* experiments, and it can be represented as M*N matrix. It presents different gene expression levels *X*_*ij*_ (*i* ∈ *M*, *j* ∈ *N*) in this matrix. Gene expressions either overexpressed or underexpressed can be revealed in terms of two colored channel in the microarray data representing the intensity of the different developmental stage. The gene expression ratios were calculated as the median value of the pixels minus background pixel median value for one-color channel divided by the same for the other channel. We extracted the median value of the log base 2 of each gene among experimental dataset because the mean value of the normalized ratio is much easier to be affected by noise than the median value. Although microarray can be used to detect thousands of genes under a variety of conditions, there are still many missing values in microarray. The reasons for missing values include insufficient resolution, image corruption, and dust or scratches on the slide. If a gene contains many missing values in experiments, it is not easy to determine a precise expression value for each gene that causes a difficulty in the subsequent analysis of networks. However, we cannot simply remove all gene data that contains missing values because the number of remaining genes will become too small to predict the weight of interaction correctly. In order to get a better result, the genes that contain less than 20% entries missing in all experiment are picked. In order to get as complete data as possible, we use the K nearest neighbors (KNN) algorithm [13] to estimate the missing values. The gene expression levels of the genes might not always maintain the same expression in different microarray data. We choose a higher ratio between the overexpression and underexpression from microarray data to determine the expression level of the gene.

Current public protein-protein interaction (PPI) databases provide rich information, and they mostly differ on the way they acquire or validate their data. For example, human protein reference database (HPRD), Biomolecular Interaction Network Database (BIND), molecular INTeraction database (MINT), and mammalian protein-protein interaction database (MIPS) are manually curated; this means a team of biologists check the literature to find new interactions, and once an interaction is confirmed it is added to the database. On the other hand, database of interacting proteins (DIP) and European Bioinformatics Institute molecular interaction data (IntAct) are based on literature mining, and they achieve these using computational methods that retrieve the interaction knowledge automatically from published papers. Prieto and De Las Rivas have shown a limited intersection and overlap between the six major databases (BioGRID, BIND, MINT, HPRD, IntAct, DIP) [14]. The information contained in these databases is partly complementary, and the knowledge of the protein interactions can be increased and improved by combining multiple databases. Bio interaction resource (BioIR) integrates the major publicly available databases that contain protein-protein interaction information. The integrated PPI data warehouse included those databases we mentioned, and we successfully gathered 54283 available and nonredundant PPI pairs among 10710 proteins [15]. Notably, even the union of all databases is still incomplete with many unknown components and pathways of the human proteome. Then, we map genes in microarray data according to the BioIR database and extract their corresponding undirected protein interaction networks where the node set *V* represents protein *v* (*v* ∈ *V*) and edge set *E* represents the protein interactions *e* (*e* ∈ *E*) in the network.

In the randomized color-coding method, it requires a predefined pathway length. The length of the path is dependent on the execution time that they can run in practical use. In the scale-free properties of the biological network, there are minimum number of steps from a given genes to the other genes, and it means that a given gene grows rapidly which suffer from the “small world” effect [16]. According to the reason, we apply the heap-based Dijkstra's algorithm [17] for each node to get the longest shortest path of all pairs of nodes in the network. This information shows if any pair of nodes in the network can link to others at most this length. So, we use the number of the longest shortest path to be the maximum number of the length that we can search in the path. In our method, the fixed length of the path is not defined by the users and it is dependent on the topology of the protein network deposited in the protein interaction database. 

We assume that a protein interaction participates in a signaling pathway and the genes producing the associated proteins should be coexpressed and might be coregulated. Grigoriev showed that biologically relevant interacting proteins have high mRNA expression correlations [18] and the correlation of the expression genes provides some evidences and biological needs for the produced proteins in the co-expression network linked in PPI [19]. Previous works show that proteins in the same signaling network exist simultaneously at the time of their activation and the genes encoding these proteins may be transcribed approximately at the same time. The correlation coefficient can measure the strength of a linear relationship between two expression values of the gene in the microarray data. So, we applied Pearson correlation coefficient for every pairwise relation in the set of (*N* ^2^ − *N*)/2 pairs of *N* genes, and the range of the value would be [−1, +1]. In general statistical usage, the positive value in Pearson correlation indicates an increasing linear relationship, and negative value indicates a decreasing linear relationship. While the correlation is closer to +1 or −1, it denotes the perfect linear relationship between pair of genes. On the other hand, the correlation approaches to zero, and there would be little or no association among the pair of genes. We take the absolute value of correlation value to capture inhibitory activity (negative correlation) as well as activation activity (positive correlation). Then, we assign the interaction weight value of an edge as a negative logarithm of the value computed by the Pearson correlation from microarray data. Namely, we assign a weight *w*(*e*) for each edge *e* from protein *u* to protein *v* and the weight is calculated as *w*(*e*) = *w*(*u*, *v*) = −log(corr(*u*, *v*)). The negative logarithm makes the larger correlation score become of smaller weight and so on. We set the pathway identification problem is a minimum-weight linear path searching problem.

### 2.2. Color-Coding Methods Based on Characteristics of Network Topology

All possible paths in protein-protein interaction network should be ideally scanned to find the best path with the minimum weight. However, to enumerate and search a linear pathway with weights in the protein-protein interaction networks where each node occurs at most once a computationally intractable for realistic-size problems [6]. The idea of color-coding technique is to apply randomly *k* colors to proteins in the networks and search only those paths under some colored constrains in order to decrease the computational time in comparison with an exhaustive search. However, randomly chosen proteins with *k* colors may fail to detect pathways if any two neighbor nodes own the same color. Randomly colored method need to ensure that the desired or important proteins have different colors with its neighbors. Due to scale-free connectivity distribution of the biological network and “small world” effect that facilitates fast propagation to communicate with other genes, high degree proteins, called hubs, tend to connect with each other and may play an important role in regulatory events, and articulation points also play crucial roles in biological network since they tend to hold and maintain the functional communicating capabilities among proteins [16]. According to the characteristics of biological networks, we wish to detect the nodes with biological meaning using the following color-coding techniques.

1. Scale-free networks have a few nodes with a very large number of links, and many nodes have only a few links [16]. The degree of a node in a graph is the number of edges adjacent to the node. Given limited colors, high-degree node should have a different color with its directed neighbor proteins. On the contrary, the node with single link called the leaf of a network can have the same color with its adjacent protein if unavoidable.
2. Articulation points are critical vertices for communication in the network. The functional pathways should pass through the articulation point to transfer functional signals. The graph would fall into many connected components and become disconnected without articulation points. Therefore, for an articulation points should tend to have a different color with its directed neighbor proteins.

In the color-coding procedure, we assign every node *v* a color with the above characteristics, and the number of color is dependent on the length of longest shortest path we mentioned. First, we sorted the nodes in PPI networks by their degrees and sequentially based on their degrees to assign colors to a node that tend to have a different color with their neighbors dependent on the number of the colors we could use. If a node is already colored, we continue to search next higher degree of nodes until there is no non-color node in the graph. The node with degree 1 denotes the leaf in the network and cannot have any children below, so we can assign the same color with its neighbors. However, it is possible to have the same color for the common neighbors of high degree nodes using this method with a limited number of colors. Therefore, we must ensure that the common neighbor is an articulation node or not and give it different colors with its neighbors so that it can be detected.  Algorithm 1 shows the pseudocode of our color-coding method.

**Figure 1:**
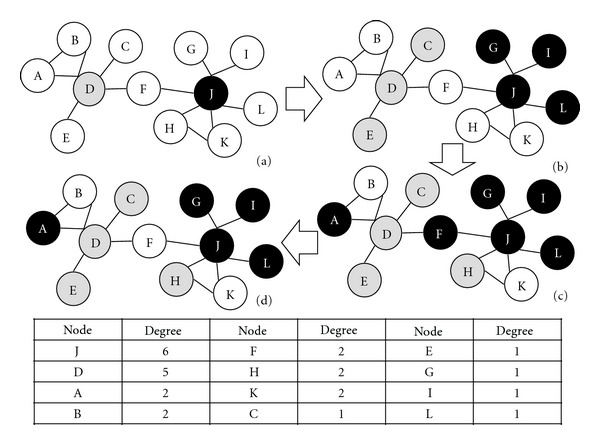
The steps of color-coding method. (a) Coloring the high-degree nodes in step  4. (b) The node with degree 1 having same color with its neighbor in step  7. (c) Randomly color the uncolored nodes in step  9. (d) Check the articulation hubs such as node *F* with different color with its neighbors in step  15.

Take an example as shown in Figure 1; it denotes the steps of color-coding method. Figure 1(a) shows randomly choose colors from white, grey, and black colors to draw the nodes with high degree based on step 4 in our color-coding method algorithm in Algorithm 1. Figure 1(b), if the node is with only one degree, can be the same color with its neighbor based on step 7. Take an example; nodes G, I, L (black), and C, E (grey) are assigned the same colors with each their hub neighbor node J and D. Figure 1(c) randomly assigns colors to the nodes that have not been colored and draw different color with hub based on step 9. Figure 1(d) check articulation hub has different color with its neighbors based on step 14–16. The articulation hubs such as node *F* colors white colors which is different with its neighbors, node D and J. 

To extract a pathway that makes biological sense beside topology characteristic, we need to take into consideration biological knowledge as constraints in order to avoid extracting biologically insignificant pathways. Therefore, we assign each protein a number *L*(*p*) of the unique cellular location based on the Gene Ontology database, and each protein can correspond to a location of intracellular, membrane, cytoplasm, or nucleus. According to the assigned location segment *L*(*p*), we extract a path from *p*_1_ to *p*_*n*_ so that the path (*p*_1_, *p*_2_,…, *p*_*n*_) for each *p*_*i*_ satisfies the partial ordering location constraints *L*(*p*_1_) ≤ *L*(*p*_2_)≤⋯≤*L*(*p*_*n*_). According to the characteristics of biological networks and nondecreasing cellular location, we can detect linear paths passing through articulation points or the high-degree nodes and also avoid extracting the paths in only one location of the cell. This restriction can reduce the complexity of the search process. 

### 2.3. A* Algorithm as Heuristic Search

Based on the color coding method to extract a colorful pathway with *k* colors, it still cost exponential time to execute with parameter *k*. In search for paths using a traditional tree search method, it may expand a large collection of new nodes while traversing new level of tree. To speed up the search procedure, it needs to prune the unexplored new nodes heuristically. We use the idea of A* search to design a pruning strategy, and the heuristic function is to determine the weight of a pathway that reflects some biological feasibility and significance to some extent. First, we defined the weight of a path as the sum of weights of edges in the path. The formula is defined as follows (1):

(1) $$w\left(p\right)=\sum_{e\in p}w\left(n\right),$$

where *w*(*e*) is the weight of a node *n* in the path.

In the preprocessing step, we determine the minimum weight of nodes in the network as *w*_min⁡_. We calculate the weights of the simple paths with the same length *l* between different start and end proteins in the uncolored network. We ran the procedure 5000 times to determine the scores of all paths in the experiments formed a normal distribution. We extract an average weight of node as *w*_avg_ and the error rate based on the standard deviation *w*_STD_ to find the optimal pathway in estimating bound heuristic function of *h*(*x*) for a node *x*. After searching a fix number of *d* in the paths, we calculate current weight of a path as function of *g*(*x*), and the overall heuristic function of *f*(*x*) is defined in (2) for finding a pathway with an optimal (minimum) weight

(2) $$f\left(x\right)=g\left(x\right)+h\left(x\right)=w\left(P_{d}\right)+w_{\min}\times \left(l-d\right),$$

where *l* means the total number of nodes in a path, *d* means the number of nodes from the starting point that we must traverse in the network, *w*(*P*_*d*_) means the weight accumulated up to the current node *x* with a length parameter *d*, and *w*_min⁡_ means the minimum weight calculated in the preprocessing experiments.

Because the lower *f*(*x*) a node is estimated, the more likely is it to be searched. So, we set a bound score for a path *p* with length *l* that is defined as follows (3) to control the quality of the path we could find:

(3) $$\text{Bound\_Score}\left(p\right)=\left(w_{\text{avg}}+\alpha \times w_{\text{STD}}\right)\times l,$$

*α* is a constant factor to control the bound, *w*_avg_ means the average weight calculated in the preprocessing experiments. *w*_STD_ means the standard deviation calculated in the preprocessing experiments.

**Figure 2:**
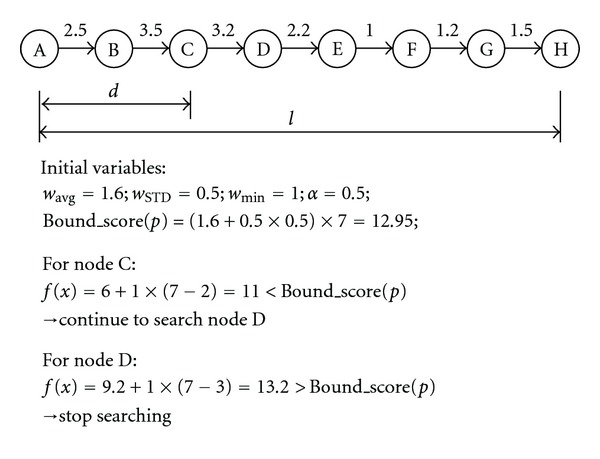
An example for A* searching method.

While we move to the next node through the edge in the each search process, we compute heuristic function *f*(*x*) and compare it with the initially set bound score. If *f*(*x*) exceeds the initially set bound score, we do not expand the node further. For the nodes that are allowed to expand, their children nodes are expanded, and their heuristic functions are computed and compared with the bound score again until the search reaches the end node. As the example in Figure 2, we consider finding a pathway with length *l* = 7 from the initial node A to the end node H.

First we explored a fix *d* = 2 from initial node A that lead us to node C; we start to estimate the weight of a path with an additional length of 5 that yields a total weight 11 from current node C. The estimated weight of the path is smaller than the bound score 12.95; therefore, we continue to traverse its children. The function of *f*(*x*) of current node D is 13.2, and therefore we cannot search into its children. Signal transduction pathway discovery is to extract the linear or tree-like path in the protein-protein interaction. Our method guarantees to find optimal path from the colorful paths in real world complex biological networks; we prove the A* heuristic function straightforward to analyze if it is used to get the optimal colorful paths with Tree-Search using admissible and monotonicity (consistency) properties based on triangle inequality [20].

**Lemma 1**

In (2), A* is optimal if *h*(*x*) is an admissible heuristic, that means *h*(*x*) never overestimates the weight of the interaction to reach the optimal path.

**Proof**

Admissible heuristics are by nature optimistic because the cost of solving the problem is less than the cost actually is. Therefore, we assign the minimum weight *w*_min⁡_ as *h*(*x*) is always less or equal than the weight of interaction actually is. The *g*(*x*) is the exact weight of the interaction we have already traversed; the *f*(*x*) with estimated weight *h*(*x*) never overestimates the true cost of an optimal path through the length *l*.

**Lemma 2**

**Figure 3:**
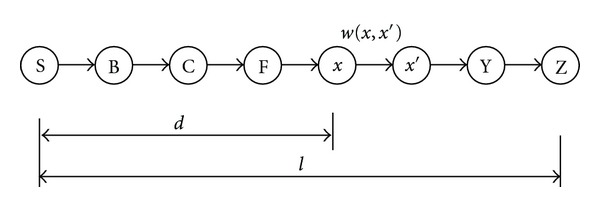
The example for consistent property of A* search.

A* is optimal if *h*(*x*) is consistent which means protein *x*′ is a mediator of protein *x* with the edge *e* and its weight is equal to the *w*(*x*, *x*′) in Figure 3. Triangle inequality in (4) stipulates that each side of the triangle cannot be longer than the sum of the other two sides. Therefore, the estimated cost of reaching the optimal path to *x* is no greater than the step cost of getting to *x*′ plus the estimated cost of reaching the goal from *x*′

(4) $$\begin{array}{cc} f\left(x\prime \right) & =g\left(x^{\prime }\right)+h\left(x^{\prime }\right)\\ & =g\left(x\right)+w\left(x,x^{\prime }\right)+h\left(x^{\prime }\right)\\ & \geq g\left(x\right)+h\left(x\right)=f\left(x\right). \end{array}$$

**Proof**

We have

(5) $$\begin{array}{cc} f\left(x^{\prime }\right) & =g\left(x^{\prime }\right)+h\left(x^{\prime }\right)=g\left(x\right)+w\left(x,x^{\prime }\right)+h\left(x^{\prime }\right)\\ & =w\left(P_{d}\right)+w\left(x,x^{\prime }\right)+w_{\min}\times \left(l-d-1\right)\\ & =w\left(P_{d}\right)+\left(w\left(x,x^{\prime }\right)-w_{\min}\right)+w_{\min}\times \left(l-d\right)\\ & \geq w\left(P_{d}\right)+w_{\min}\times \left(l-d\right)\\ & \geq g\left(x\right)+h\left(x\right)=f\left(x\right). \end{array}$$

Our assignment of the minimum weight *w*_min⁡_ as *h*(*x*) is always less or equal than all the weights of the interactions *w*(*x*, *x*′) in the network; it shows the nondecreasing value of *f*(*x*) along the path.

### 2.4. Functional Evaluation on the Pathway Detection Results

Regardless of the efficiency improvement of pathway detection, its relevance of biological function is also important to validate the methods and results. Therefore, we evaluated the biological functional enrichment of the pathways that were found by our methods. We estimated the statistical significance of the detected pathways using the Ontologizer toolkit [21]. Ontologizer uses hypergeometrics distribution to measure the quality of a pathway by comparing it with a set of randomly pathways chosen from the whole networks. For each node in a given pathway, it is annotated with a GO term in Gene Ontology. In our experiments, we used the category under “biological processing” in GO database and “Parent-Child Interaction” and “Bonferroni option” of Ontologizer to evaluate our results. The *P* value denotes the statistical significant of our results, and we assume high functional enrich pathways as those with a *P* value below significant level 0.05. Generally speaking, the proteins involved in a biological pathway tend to have similar functions or processes. Gene Ontology database annotated function and process categories of genes that can be used to check if our predicted pathways belong to the similar function or processes. That is why we show our functional enrichment pathways with Gene Ontology annotation to enhance the understanding of the functions of genes involved in the pathways.

## 3. Experiments

We implemented our methods in java programming language in order to detect possible pathways in the biological networks. Then, we evaluated the methods using two sets of test data with CPU Intel 1.73 GHz and 1 GB main memory running under the windows operating system. The first test data is the network of yeast extracted from DIP [22] database, and it contains 3789 proteins and 14707 interactions. The gene expression data download from Mega Yeast gene expression data set which contains 6348 genes and 499 yeast microarray experiments [23]. We applied our method to search for the known pathways comparing with KEGG database and previous Steffen's Netsearch [3], Scott's color-coding [6], Zhao's ILP [10], and PathFinder [11] algorithms. As a second test data, we integrated microarray data extracted from [24] that consists of 62 primary tumors in Stanford microarray database (SMD) [25] and 4767 proteins having 21878 protein-protein interactions to search for the significant pathways in human prostate cancer. The length we search for in our method is based on the longest shortest paths in whole networks which are 11 in human protein interaction network and 9 in yeast network.

### 3.1. Global Results

After following our color-coding method, there are still some nodes need to be assigned colors by random, and it may make us fail to find the colorful path in the network. Therefore, we calculate the failure rate of our method to detect the colorful path. According to the weights of all paths in the experiments formed a normal distribution, we calculate our searching scope based on the standard deviation and alpha value. The number of possible permutation of the colorful path and the number of possible coloring of vertices in the path are shown in (6) [6]

(6) $$\frac{P_{L}^{K}}{K^{L}}>e^{-K}.$$

Based on (6), we calculated the probability of the failure rate for pathway detection in (7) [6]

(7) $$p\left(\text{failue}\right)={\left(1-e^{-K}\right)}^{t}.$$

**Figure 4:**
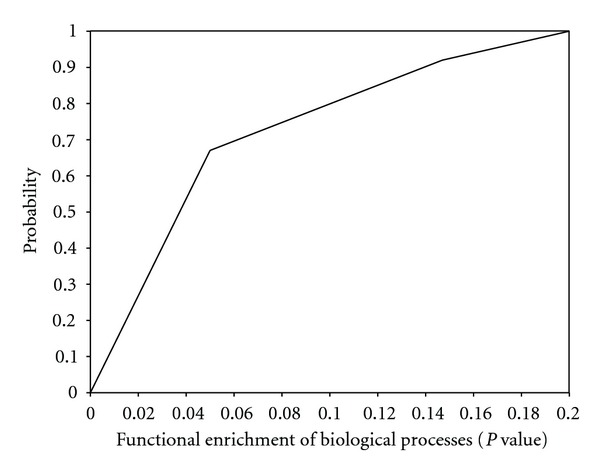
Cumulative distribution of *P* value in our detected paths. *x*-axis: *P* value, *y*-axis: percent of paths with corresponding *P* value or better.

In theory, if we let the probability of failure rate exceed the certain except value as 0.05, we should run more than 8900 of interactions of the program with length 8. Due to the failure rate of random color-coding method, the colorful paths cannot always be detected with color-coding method. We ran 10000 time to compute the paths that start between any node belonging to the membrane protein category (GO: 0005886 or GO: 0004872) and any node belong to the nucleus protein category (GO: 0005634) with varying lengths to calculate the probability of extracting colorful paths in yeast network.  Table 1 shows the successful rate between our method and randomized color-coding method, and it shows that our method has higher probability to get colorful paths than randomized color-coding method. The maximum lengths of the paths among hubs and articulation points are 4 in human protein interaction network and 5 in yeast network. It denotes that our method can color the different colors among hubs and articulation points. It makes the search procedure can be explored and also help us to get the colorful paths in the network. On the other hand, we evaluated its functional enrichment for each path we detected in yeast network in Figure 4, and it denotes that 66% of them are functional enrichment with significant biological process (*P* value < 0.05) comparing random chosen.

### 3.2. Yeast Networks

We applied the previous state-of-art methods to compare with our method with yeast network and gene expression data and test to reconstruct the three known signal pathways: a pheromone response signaling pathway, a filamentous growth pathway, and cell wall integrity pathway. The signal pathways can be described as a main chain path that contains important protein cascades. The direct interactions in KEGG pathway may be actually undirected interactions through several proteins in the protein networks. For each pathway, we use one more than the number of nodes in the main chain path as the length of our color-coding method to search the colorful path. We first determined the degrees of proteins in the yeast networks and applied the program implemented by Hyung-Joon Kim to extract the articulation points whose time complexity is *O*(*n* + *m*) in the worst case, where *n* and *m* is the number of nodes and edges in a network, respectively. The proportion of proteins with at least a degree 8 is 31.09% and 19.13% with degree 1 in yeast network. 482 articulation points are extracted from network, and the number of intersection of the articulation points and protein with a degree lower than 8 is 220.

**Figure 5:**
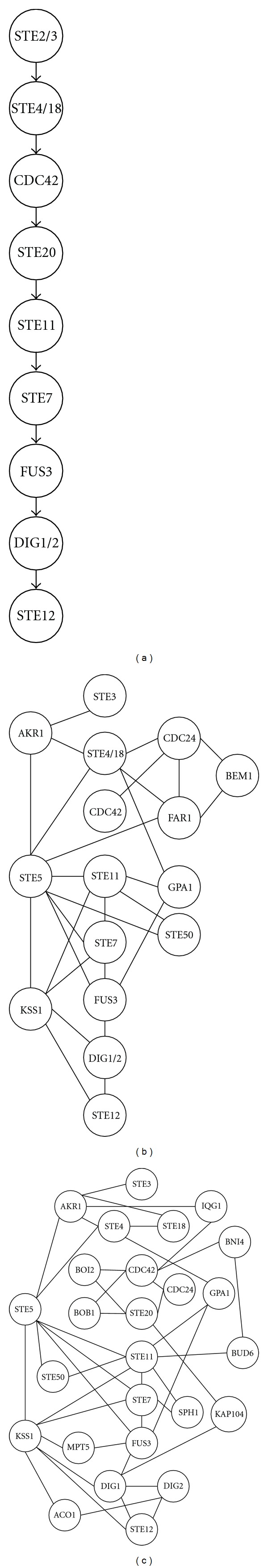
(a) The main chain path of pheromone response pathway (b) the assembly paths detected by Scott et al. method (c) the assembly paths detected by our method.

The functional purpose of a pheromone response pathway is to address which mating type a yeast cell is. The main chain of a pheromone response pathway is from the membrane protein STE3 to transcription factor STE12 which is shown in Figure 5(a).  Figure 5(b) shows the assembly networks for paths of lengths 6–10 using a random color-coding method by Scott's method.  Figure 5(c) denotes an assembly pathway of length 9 detected by our method. Both methods assemble the pathways based on the nodes that have a hit with at least half of nodes in the main chain of the pheromone response pathway. Those two methods found KSS1 which is a MAP kinase and the negative regulator, AKR1, but our method detected STE20 which is missing in Scott's results. Scott's and our results both detected CDC24 which is not found by Netsearch and may form protein complexes with FAR1, and a heterotrimeric G-protein may mediate chemotropism that is essential for morphological changes in response to pheromone during mating processes [26]. It can be seen that all the proteins in the main chain are covered by our method. However, we failed to detect FAR1 and BEM1 comparing to Scott's method. The failure of detecting FAR1 because the paths are involved with those two proteins did not have a hit with at least half of nodes in the main chain of the pheromone response pathway. In our results, we detected BOI2 which appear in the same protein complexes with BEM1 founded by MIPS database [27]. MPT5 and SPH1 are also found by Steffen's Netsearch toolkits, and STE50 is detected by Zhao's ILP method. We also detected 7 new proteins (IQG1, BNI4, BUD6, BOB1, BOl2, KAP104, and ACO1) related to pheromone response. Most of them are sensitive to pheromone response and sexual differentiation. We found that BOI2 and BOB1 which can bind to SH3-2 domain of BEM1 are related to pheromone-responsive mitogen-activated protein kinase cascade [28]. KAP104 is critical for negative regulation of the pheromone response [29]. The previous research [30] denotes that the effects of CDC42 and IQG1 on the pheromone response pathway promote cytokinesis with AKR1. Although it is difficult to confirm in vivo that all proteins are exactly involved in the pathway, we use Gene Ontology to verify the proteins if they have the reasonable biological functions and processes in the pathways. As a final test to evaluate our results, the paths we detected presented high statistical significance for the functional enrichment of “response to pheromone” category with *P* value below 0.05.

**Figure 6:**
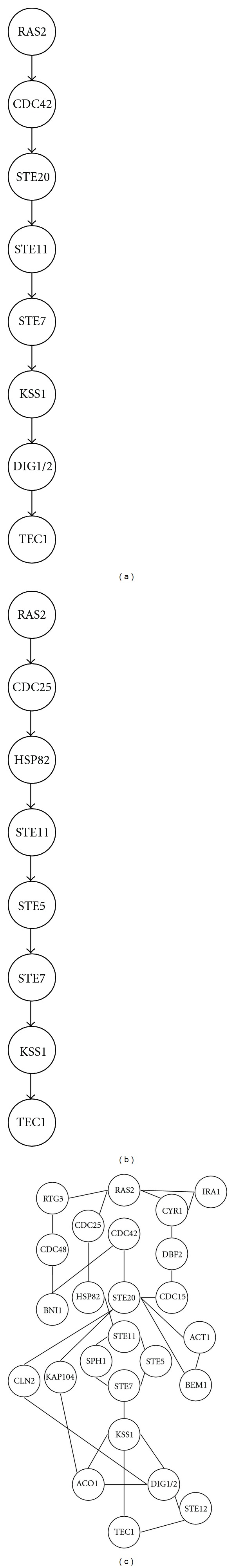
(a) The main chain path of filamentous growth pathway (b) the main chain detected by Scott et al. method (c) the assembly paths detected by our method.

The main chain of the filamentous growth pathway from RAS2 to TEC1 is shown in Figure 6(a). The main chain detected by Scott's is shown in Figure 6(b), and the assembly paths detected by our method contain 26 proteins in Figure 6(c). Both methods can detect the path through CDC25 and HSP82 protein to form a main chain of the filamentous growth pathway due to the fact that there is no direct link between RAS2 and CDC24 in Figure 6(c). Then, we compared the network-generated previous works but we do not detect ABP1, COF1, LAS17, BUD6, and SRV2 which are found by Zhao's ILP method, because we extract protein BEM1 that are known to be involved in the function of actin filament organization and form the same protein complexes with those proteins [27]. RAS2 is regulated by GAPs IRA1 and activates to increase the cyclic AMP (cAMP) concentration. CDC15 kinase activates a protein kinase complex consisting of the kinases DBF2 [31]. BNI2 is the potential effectors of CDC42 in yeast [32]. The actin protein ACT1 displayed two-hybrid protein interactions with BNI1 and repress filamentous differentiation in response to low nitrogen [33]. The expression of CLN2 also enhances the filamentous growth [33]. 

**Figure 7:**
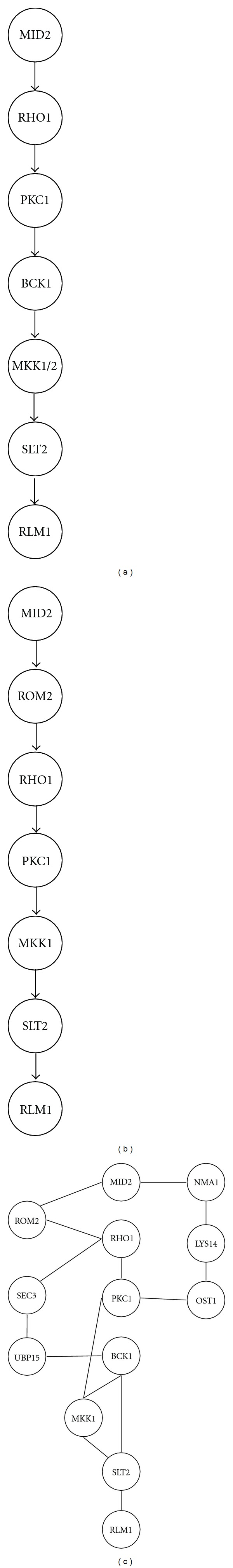
(a) The main chain path of cell wall integrity pathway (b) the main chain detected by Scott et al. method (c) the assembly paths detected by our method.

The cell wall integrity pathway from MID2 to RLM1 with path length 8 is shown in Figure 7(a), and this signaling pathway is critical for cell expansion during growth. The main chain detected by Scott's method is shown in Figure 7(b), and our method can identify the known pathway well as shown in Figure 7(c). It shows that cell wall signaling is controlled by the small G-proteins of GTPases, RHO1 that interacts with the action of guanosine nucleotide exchange factors such as ROM2 [31]. PKC1 interacts with the unit of oligosaccharyl transferase (OT) complex, OST1. SEC3 is a spatial landmark for the exocyst to the site of exocytosis independently of polarized secretion [34]. We found that our methods provide richer information consistent with current researches and also detect new related proteins. The proteins detected by previous and our methods in the yeast pheromone signaling, filamentous growth, and cell wall integrity pathways are shown in Supplemental Table S1 available online at doi: 10.1100/2012/315797. The results show that the number of proteins in the main chain we detected is higher than other methods, and other new proteins are validated by published papers and experiments. 

Due to the incomplete interactions there is no gold standard which means that the true signaling network is not available now. To see the performance of the different methods, we applied the same test set used in the previous works in terms of the precision, and recalls were employed in this work. The precision is defined as the percentage of the proteins detected by the computational methods that are also in the KEGG main chain pathway. The recall is the percentage of the proteins in the KEGG main chain pathway that are also detected by the computational methods. Due to the incomplete of knowledge of the signaling network, we also apply *F*-measure to evaluate the performance, and this value is not dependent on the absolute match value but only comparative measures. It is defined as 2  ∗ precision ∗ recall/(precision + recall).  Table 2 compares our method and previous methods with respect to precision, recall, and *F*-measure. In the pheromone signaling pathways, PathFinder had 81.8% (9/11) recall and 56.3% (9/16) precision in recovering the pathways. Scott's color-coding method had 90.9% (10/11) recall and 55.6% (10/18) precision whereas the NetSearch prediction had 72.7% (8/11) recall and 42.1% (8/19) precision and ILP method had 100% (11/11) recall and 32.4% (11/34) precision. Our method had 100% (11/11) recall and 42.3% (11/26) precision. ILP and our proposed methods get the highest recall and we also have higher precision than ILP. It shows that we can detect all the proteins that have been known in the pathways without extracting more nonrelated proteins. In the filamentous growth pathways, Scott's color-coding method only detects 5 proteins in the main chain of the path. Pathfinder and our methods detect the maximum number of proteins involved in the main chain comparing with the other methods, but we get highest precision and *F*-measure. In the cell wall integrity pathways, we also get the higher precision and recall than the previous works. Since the true genes in the signaling networks are not actually known. Although it is difficult to know exactly correct genes in the signaling network, those genes may have casual relationships and are also reported in published papers. We can learn that our proposed method performs comparably well with previous existing methods in the yeast signaling pathways deposited in KEGG database. 

### 3.3. The Significant Pathways of Prostate Cancer

Prostate cancer is a leading cancer and aggressive metastasis disease worldwide, and it is the second common cancer death among men [35]. Although numerous studies use microarray analysis to identify the individual genes during the disease processes, the important pathway remain unclear. In microarray data preprocessing, it consists 44,014 genes from microarray data after combining redundant gene. When we remove the genes that have more than 20% missing values in the microarray dataset, we extract 11,130 genes and imputed 7,588 number of data with KNN method. We evaluated the KNN method for imputing missing values in the microarray data. First, we deleted 1,750 origin values at random one by one to create test data sets and estimated the missing value to compare with the origin values. The accuracy of estimation values are calculated by root mean squared error (RMSE) which denote the different values between imputed values and original values, divided by the number of missing value we computed. The lower RMSE denotes higher accuracy for estimating the missing values. While setting the 11–15 nearest neighbors in KNN method, we could get the lowest error rate. According to the performance of KNN data impute algorithm in Supplemental Figure S1, we extract 15 neighbor genes which caused the lowest RMSE rate with 20% missing values in the microarray dataset. 

**Figure 8:**
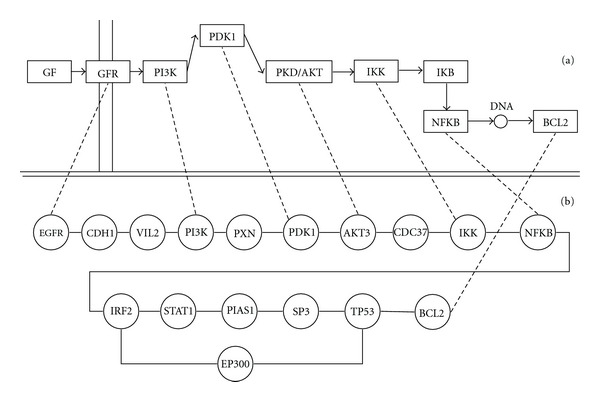
(a) One of the KEGG prostate cancer pathways (b) the paths detected by our method. The dash line means the mapping genes.

The proportion of proteins with at least a degree 8 is 29.56% and 19.46% with 1 degree in the prostate cancer network. 639 articulation points are extracted from network, and the number of intersection of the articulation points and protein with a degree lower than 8 is 231. If we use the random color-coding method with limited color, it may get same color between articulation point and its neighbor using random color-coding method and lose a lot of biological meaning for pathway detection. That is the reason why we modify the traditional random color-coding method into the current one. Interestingly, several reports suggested that the degree of a node tends to be high in the networks if the protein represents a primary factor and protein mutation in the cancer cell, and the articulation points that cause a disruption in the network also tend to be related to cancer mutation [35]. The conclusion supported our color-coding methods based on the degree property of a network, and it provides an efficient way to search a pathway that contains those highly biologically significant proteins. We successfully reconstruct the paths from EGFR to BCL2 in KEGG pathway database in Figure 8 to validate our results. Figure 8(a) shows the one path in prostate cancer pathways, and Figure 8(b) shows the paths reconstructed from our method, and the dash line denotes the correspondence between the same genes between two paths. We can map genes in the prostate cancer signal pathway in KEGG database with more detail interactions except IKB that did not have a hit with at least a half of nodes.

As an example from membrane protein EGFR to BCL2 with length 15 in Supplemental Figures S2 and S3, we assembled network extracted from our method to explain the potential reaction pathway in human prostate cancer network. We evaluated our results related to prostate cancer with public Online Mendelian Inheritance in Man (OMIM) National Center for Biotechnology Information (NCBI) [36], KEGG pathway database [37], PGDB database [38], and published papers. In particular, all paths we detected are 100% functional enrichment exceeding random expectation and belonging to the high significant cell cycle biological processes (*P* value =  5.668*e*−6), and prostate cancer included the PIK3 pathway. The grey color nodes denote cancer-related genes annotated in databases and those genes belong to high-degree nodes or articulation hubs. For example, gene BRCA1 with degree 129 is strongly related to prostate cancer, and articulation hub of gene NCOA4 with only degree 8 is also the annotated cancer gene in prostate cancer. For a part of the network in Supplemental Figures S2 and S3, we discovered that protein AR is annotated in KEGG database as oncogene in the prostate cancer pathway, and papers [39, 40] also support the disease-related proteins in prostate cancer growth via TP53 mechanism. MYC, BRCA1, BRCA2, RNF14, CCND1, ATM, ESR1, NCOA4, SMARCA4, PCNA, SMAD2, CAV1, and RB1 are annotated the prostate cancer-related genes, and CDH1 is mark gene as tumor suppressors in PGDB database. The evidence for protein NR3C1 related to prostate cancer and MAPK proteins, member of the MAP kinase family, involved in the cellular processes such as apoptosis, cell proliferation, and it also leads to G1 cell cycle arrest in prostate cancer [41]. BRCA1 and BRCA2 proteins play important role in DNA repair in both S and G2 checkpoint phase of the cell cycle. Except the genes discovered by the public database, we list the genes that are extracted by our method in Supplemental Table S2 and those results we detected confirmed by present biological evidence from the literature. 

### 3.4. Execution Time of Our Methods

**Figure 9:**
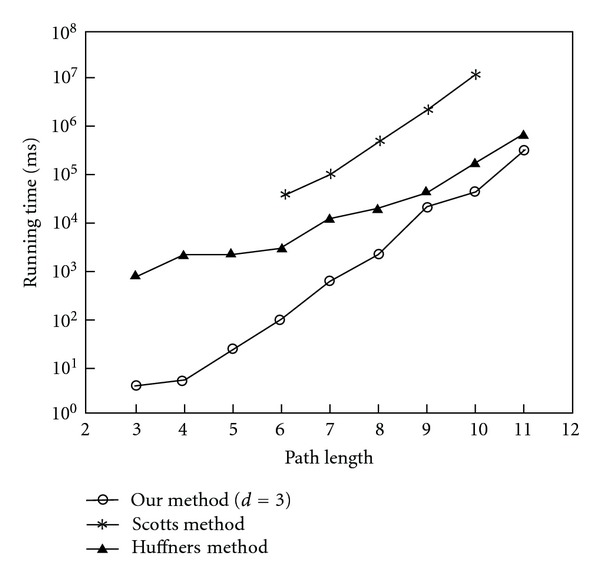
The comparison of runtime in human prostate cancer networks between Scott's, Huffner's method, and our methods.

We detect that the length of 11 is the longest shortest path in our prostate cancer network, so we try to detect the path with the length from 3 to 11 as our experiment testing. Based on the same protein-protein interactions and the weights of interactions, we ran the Scott and Huffner's work to compare with our method. We randomly choose the start protein that belongs to membrane protein category (GO: 0005886 or GO: 0004872) and end protein that belongs to the nucleus category (GO: 0005634). Comparing with the execution time, our method is faster than Scott color-coding method and we are able to detect the length of paths above 10 within 29 seconds while it took about 3 hours in Scott's method as shown in Figure 9. Scott's and Huffner's work applied dynamic programming in the color-coding method that still takes more time to execute in comparison with our method. Based on color-coding method to extract a colorful pathway of length *k*, it still costs exponential time to execute with parameter *k*. Our method with the pretraversed length *d* = 3 can detect the optimal colorful path under color-coding method. It may take a lot of time to detect paths only dependent on our color-coding method because we allow more candidate paths passing through the high degree and articulation hubs than previous random color-coding method. The user set parameter of alpha value let our method not only detects the optimal path but also can find the top paths that are still statistical and biologically significant.

**Figure 10:**
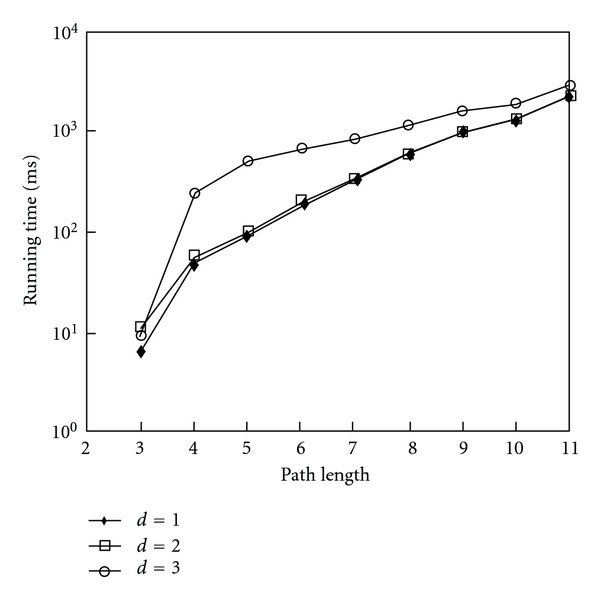
The comparison of runtime with different parameters *d*'s for pathway detection from proteins HLA-B to YWHAZ.

The A* procedure with pretraversed length *d* = 3 presents an efficient way to extract longer lengths of paths. Therefore it is important to filter the higher weights of paths and keep looking for lower weights of paths in the search procedure. To consider the effect of parameter *d*, we implemented the experiments to discuss the runtime time between different path lengths from proteins HLA-B to YWHAZ against different pretraversed lengths *d* in A* searching procedure. The results are shown in Figure 10, and it denotes faster execution time with a shorter length of parameter *d* because it filtered out a large number of nodes to be expanded early. 

## 4. Conclusion

We developed methods to detect pathways with a color-coding technique which takes into consideration the characteristics of biological network topology and also speeds up the search with an A* heuristic method. We are able to reconstruct the yeast with the same known signaling pathway as previous studies but we also showed the potential to detect new functional related pathways as well. Based on the degree and articulation points, it ensures that those biological significant proteins can be found in the colorful path instead of merely random coloring. Purely A* search with traditional random color-coding method may extract paths without passing through articulation points or the high-degree nodes that might contain biological meanings. This approach is able to efficiently integrate microarray data and protein-protein interactions for the signal pathway detection of cancer. We are able to detect the proteins and their pathway related to prostate cancer by recent database and published papers. The merit of this research would help biologists to understand the cellular mechanism more easily. In the future, we can study the effects of other properties of network topology such as centroid and closeness with color-coding method. However, PPI database consists of interaction pairs under different physiological conditions that may not exist in all tissues, and it is also not complete such as phosphorylation interactions. In the future, we could use different microarray data about the cancers to test our methods and integrate further the protein-DNA interaction information to understand the reaction pathways of upstream and downstream biological interaction mechanisms response to various diseases. The edge orientation of the interaction of proteins may also be a problem in pathway detection. We can develop the computational analytic tools for detecting pathway that can provide more complete and in-depth information implied in the dataset for physicians so that they could either based on it conduct further clinical diagnosis or verification. It is also a useful idea to develop a pathway query system that allows users to set conditions on their interested proteins.

## Supplementary Material

We compared our method to the previous stat-of-art methods and reconstruct three known signal pathways in KEGG database: a pheromone response signaling pathway, a filamentous growth pathway and cell wall integrity pathway. The proteins in three pathways detected by previous and our methods are shown in Table S1.
In order to deal with missing values in microarray data, we evaluated K-nearestneighbors (KNN) algorithm to determine the precise expression values. First, we deleted 1,750 original values at random one by one to create test data sets and estimated the missing value to compare with the original value. The accuracy of estimation values are calculated by Root Mean Squared Error (RMSE) with different numbers of neighbors which are shown in Figure S1.
In the experiments, we tested our method to identify prostate cancer-related networks from EGFR to BCL2 with length 15 and the results are shown in Figure S2 and S3. Table S2 denotes proteins in those networks which are confirmed by biological evidences from the public literature and databases.

## Figures and Tables

**Algorithm 1:**
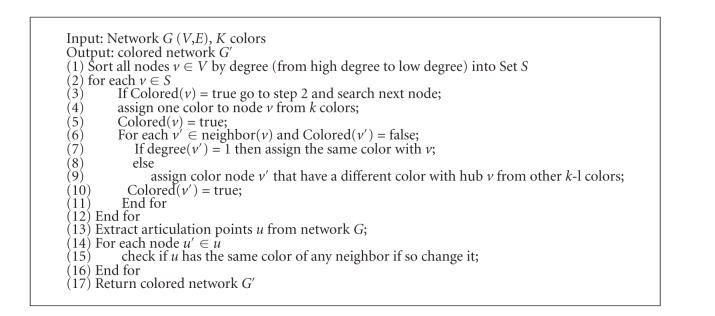
The pseudocode of the color-coding method.

**Table 1:** The successful probability with varying lengths and fixed alpha = 0.5.

Path length	Our color-coding method	Randomized color-coding method
7	97%	82%
8	97%	89%
9	99%	92%
10	99%	93%

**Table 2:** Recall and precision of previous and our methods.

Pathways	Method	Recall (%)	Precision (%)	*F*-measure
Pheromone signaling pathway	Our method	100	42.3	0.59
	Pathfinder	81.8	56.3	0.67
	Color-coding	90.9	55.6	0.69
	ILP	100	32.4	0.49
	NetSearch	72.7	42.1	0.53

Filamentous growth pathway	Our method	100	34.6	0.51
	Pathfinder	100	27.3	0.43
	ILP	88.9	28.6	0.43
	NetSearch	77.8	31.8	0.45

Cell wall integrity pathway	Our method	87.5	53.8	0.67
	ILP	87.5	38.9	0.54
	NetSearch	87.5	36.8	0.52
