# Supplementary material for: Pathway Detection from Protein Interaction Networks and Gene Expression Data Using Color-Coding Methods and A∗ Search Algorithms
Source: ScientificWorldJournal. 2012 Apr 1;2012:315797. doi: 10.1100/2012/315797 (PMC3346698; doi:10.1100/2012/315797)
Supplement: Supplementary file 1 — We compared our method to the previous stat-of-art methods and reconstruct three known signal pathways in KEGG database: a pheromone response signaling pathway, a filamentous growth pathway and cell wall integrity pathway. The proteins in three pathways detected by previous and our methods are shown in Table S1. In order to deal with missing values in microarray data, we evaluated K-nearestneighbors (KNN) algorithm to determine the precise expression values. First, we deleted 1,750 original values at random one by one to create test data sets and estimated the missing value to compare with the original value. The accuracy of estimation values are calculated by Root Mean Squared Error (RMSE) with different numbers of neighbors which are shown in Figure S1. In the experiments, we tested our method to identify prostate cancer-related networks from EGFR to BCL2 with length 15 and the results are shown in Figure S2 and S3. Table S2 denotes proteins in those networks which are confirmed by biological evidences from the public literature and databases. [file 315797.f1.pdf]

Supplemental data

Table S1 the proteins detected by previous and our methods in the yeast pheromone signaling, filamentous growth and cell wall integrity pathways (the bold words denote the overlapped proteins in KEGG main chain pathway)

Pathway name	Scott's color-coding	Steffen's Netsearch	Zhao's ILP	Pathfinder	Our method
Pheromone signaling	**STE3,STE4,STE18, CDC42, STE11, STE7,FUS3, DIG1,DIG2,STE12**, AKR1,STE5, KSS1, CDC24, BEM1, FAR1,GPA1, STE50	**STE3**, **STE18, STE4**, **STE11**, **STE7, DIG1, DIG2, STE12**, STE5, FUS3, KSS1, AKR1 , CDC24,FAR1,BEM1, GPA1, SST2,MPT5, SPH1	**STE3, STE4, STE18,CDC42, STE20, STE11, STE7, FUS3, DIG1, DIG2, STE12,** CDC24, STE50, BEM1**,** CLA4, CDC28,STE5,FAR1, MPT5,AKR1,KSS1, CLN2, CLN1, IQG1, GCS1, ACT1, SWE1, BOI1, LAS17, RSR1, GIC1, BNI1, GIC2, SPA2 (λ= 0.5)	**STE3,STE4,STE20, STE11,STE7,FUS3, DIG1, DIG2, STE12,** KSS1, STE5, BEM1,GPA1**,** CDC24, SST2, FAR1	**STE3, STE4, STE18,CDC42, STE20, STE11, STE7, FUS3, DIG1, DIG2, STE12,** AKR1, STE5, STE50, KSS1, ACO1, MPT5, IQG1, BNI4, CDC24, GPA1, BUD6, SPH1, KAP104, BOI2, BOB1
Filamentous growth	**RAS2**, **STE11**, **STE7, KSS1**,**TEC1,**CDC25, HSP82,STE5	**RAS2**, **STE11, STE7**,**KSS1**, **TEC1,DIG1,DIG2,** SRV2,ACT1 ,FUS1,FUS3,	**RAS2**,**STE11,STE7**, **KSS1**,**TEC1, DIG1**, **DIG2, STE20**, FUS3, STE5,BEM1,BUD6,SRV2,ACT1,CYR1,	**RAS2,CDC42, STE20, STE11, STE7, KSS1, DIG1, DIG2, TEC1,** STE12, FUS1,	**RAS2**, **CDC42**, **STE20**, **STE11**, **STE7**, **KSS1**, **DIG1**, **DIG2,TEC1,** CDC25,HSP82,RTG3

		STE12,CYR1,ABP1, BEM1, BNI1,SPA2, STE5,CDC25,HSP82, HSC82,BUD6	LAS17, PFY1, BNI1, STE20, GPA1, SPH1, AKR1, MCM1, ARG81, SPA2, SRV2, HSC82, ABP1 (λ= 0.5)	LAS17, CDH1, CDC7, CDC28, SPA2, BEM1, GPA1, STE4, SSK1, SWE1, SSK2,NBP2, CLA4, CDC24, STE5, PBS2, PTC1, HOG1, FUS3, FAR1, FLO8, BUD6	,STE5,CDC48, BNI1, SPH1, KAP104, CLN2, ACO1, STE12,IRA1, CYR1,DBF2, CDC15,ACT1, BEM1
Cell wall integrity	**MID2**, **RHO1, PKC1, MKK1, SLT2, RLM1,** ROM2,	**MID2**,**RHO1**, **PKC1 , BCK1**, **MKK1, SLT2, RLM1,**FKS1,RGA1, RDI1, BNI1,CDC42,GIC2, ACT1, BUD6,SPA2, SPH1,MKK2, SMD3	**MID2**, **RHO1, PKC1**, **MKK2, MKK1, SLT2, RLM1,** ROM2, RDI1, BEM4, RGA1, CDC42, BNI1, SPA2, SWI6, SWI4, SKN7, MBP1 (λ= 0.15)		**MID2, RHO1**, **PKC1**, **BCK1, MKK1, SLT2, RLM1,** ROM2, SEC3, UBP15, NMA1, LYS14, OST1

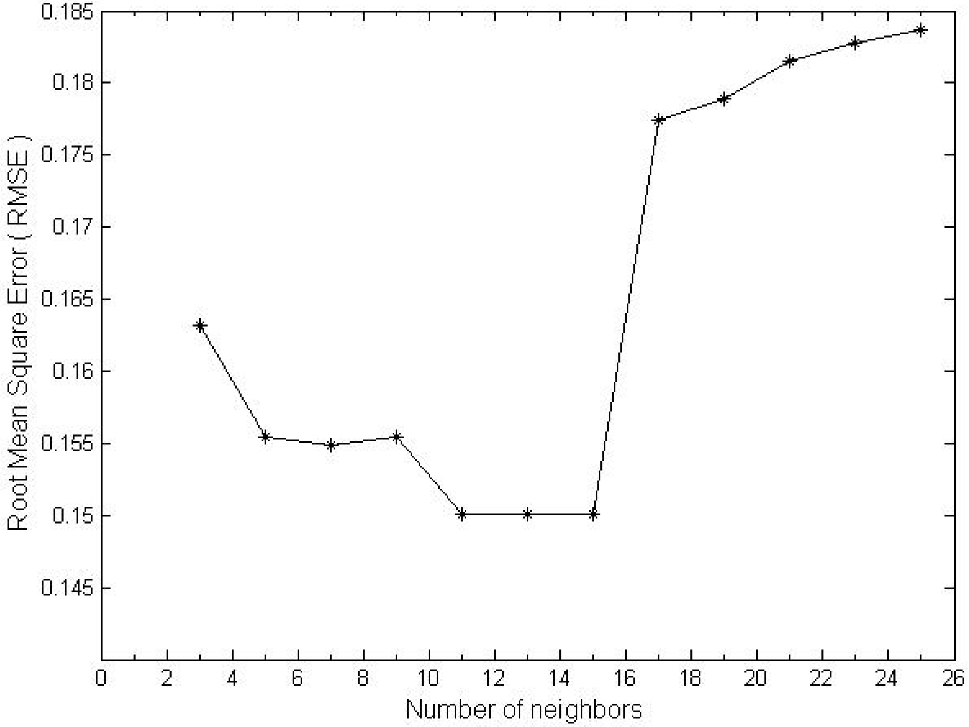

Figure S1 - effect of number of nearest neighbors used in KNN method for imputing missing values.

The accuracy of estimation values in the microarray data are calculated by Root Mean Squared error (RMSE) which denote the different values between imputed values and original values, divided by the number of missing value we computed. It shows that the estimated values with RMSE rates are under 0.185 while setting the 11-15 nearest neighbors in KNN method.

Table S2 - the evidences of the results we detected

We list the genes that extracted by our method and those results also confirmed by present biological evidence from the public literature.

Symbols	Gene name	Ref
PIK3R3	phosphoinositide-3-kinase, regulatory subunit 3	[42]
PIK3R1	phosphoinositide-3-kinase, regulatory subunit 1	[43]
FHL2	four and a half LIM domains 2	[44]
ABL1	c-abl oncogene 1, receptor tyrosine kinase	[45]
BRE	brain and reproductive organ-expressed	[46]
C11ORF30	chromosome 11 open reading frame 30	[47]
CBX5	chromobox homolog 5	[48]
XRCC6	X-ray repair complementing defective repair in Chinese hamster cells 6	[49]
PCAF	K(lysine) acetyltransferase 2B	[50]
RNF14	ring finger protein 14	[51]
APEX1	APEX nuclease 1	[52]
HDAC1	histone deacetylase 1	[53]
HSPA8	heat shock 70kDa protein 8	[54]
SMAD3	SMAD family member 3	[55]
SP1	Sp1 transcription factor	[56]
CCND1	cyclin D1	[57]
LRPPRC	leucine-rich PPR-motif containing	[58]
YWHAZ	tyrosine 3-monooxygenase/tryptophan 5-monooxygenase activation protein	[59]
GTF2B	general transcription factor IIB	[60]
PSMC2	proteasome 26S subunit, ATPase, 2	[61]

GTF2H1	general transcription factor IIH	[62]
CDK7	cyclin-dependent kinase 7	[63]
NCOR2	nuclear receptor co-repressor 2	[64]
SET	SET nuclear oncogene	[65]
FANCD2	Fanconi anemia, complementation group D2	[66]
PPP2CA	protein phosphatase 2, catalytic subunit, alpha isoform	[67],[68]
CEACAM1	carcinoembryonic antigen-related cell adhesion molecule 1	[69]
ANXA2	annexin A2	[70]
VCL	vinculin	[71]
PTPN11	protein tyrosine phosphatase, non-receptor type 11	[72]
STRN4	striatin, calmodulin binding protein 4	[73]
CALM1	calmodulin 1	[74]
BCR	breakpoint cluster region	[75]
JAK1	Janus kinase 1	[76]
STAT3	signal transducer and activator of transcription 3	[77]
DDX5	DEAD box polypeptide 5	[78]
H2AFX	H2A histone family, member X	[79]
PIAS1	protein inhibitor of activated STAT, 1	[80]
SP3	Sp3 transcription factor	[81]
GABPA	GA binding protein transcription factor, alpha subunit 60kDa	[82]
BAG1	BCL2-associated athanogene	[83]
UBE3A	ubiquitin protein ligase E3A	[84]

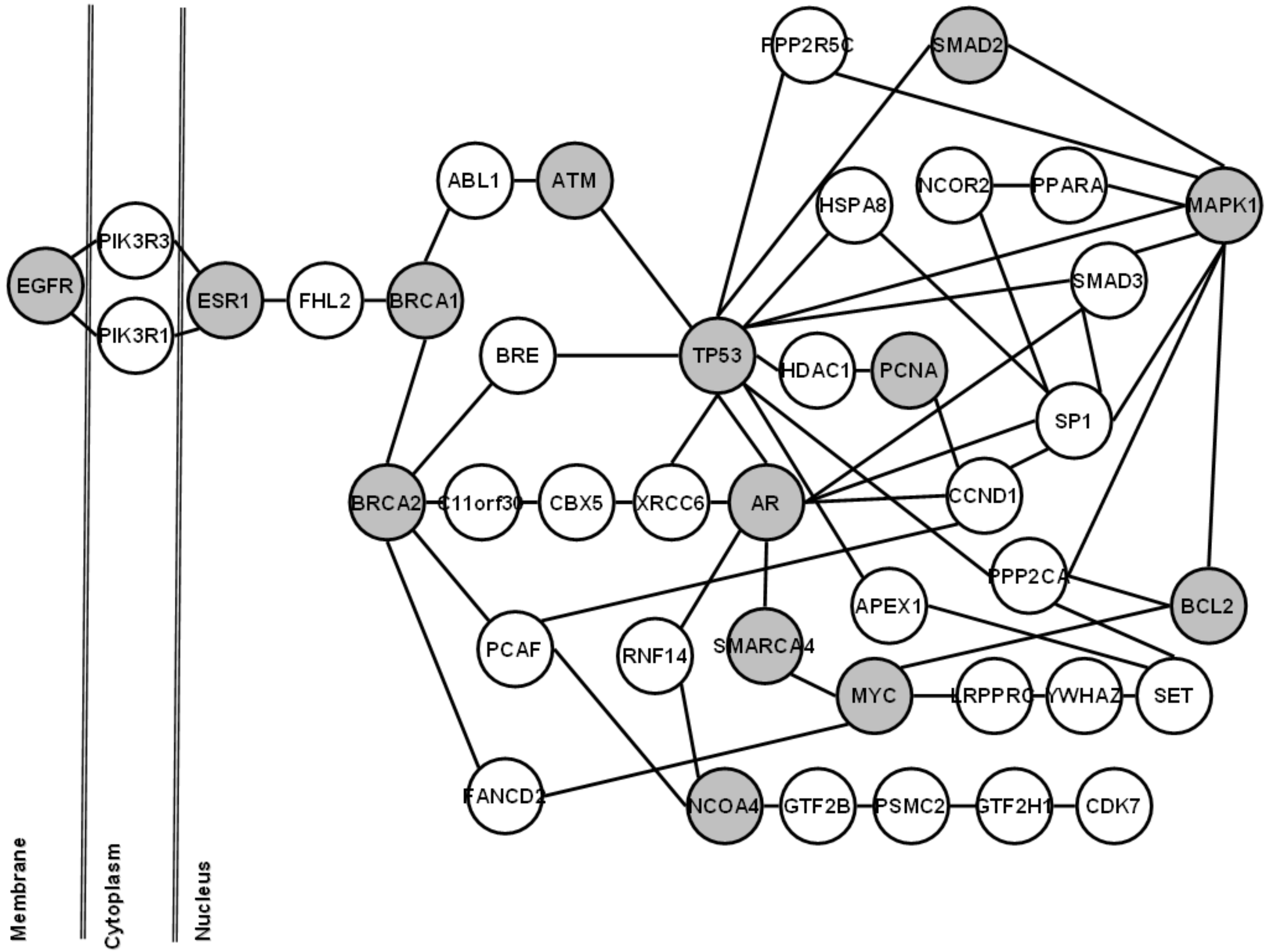

Figure S2 - the part of networks of human prostate cancer from EGFR to BCL2; The nodes with grey color denote the disease-related genes

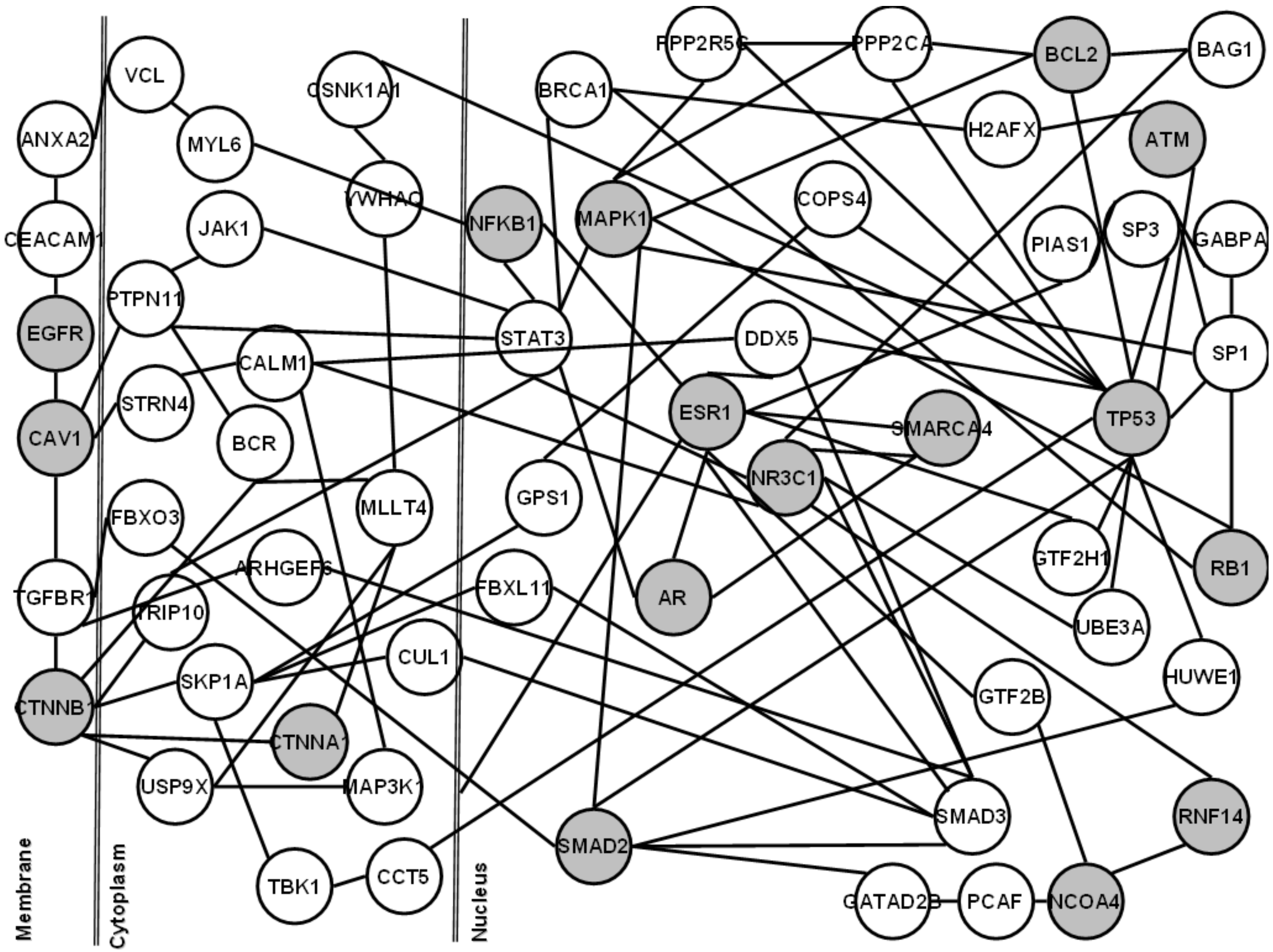

Figure S3- the part of networks of human prostate cancer from EGFR to BCL2; The nodes with grey color denote the disease-related genes

In Figure S2 and S3, we search the significant paths from membrane protein EGFR to BCL2 with path length 15 and assembled the paths to human prostate cancer network.

Reference

[42] Zhang L, Huang J, Yang N, Greshock J, Liang S, Hasegawa K, Giannakakis A, Poulos N, O'Brien-Jenkins A, Katsaros D, Butzow R, Weber BL, and Coukos G: **Integrative Genomic Analysis of Phosphatidylinositol 3′-Kinase Family Identifies PIK3R3 as a Potential Therapeutic Target in Epithelial Ovarian Cancer**, Clinical Cancer Research., 2007, **13**:5314.

[43] Paradis A, Kantoff P, Giovannucci E, Stampfer M, and Ma J: **Association between the Met326Ile polymorphism of the p85α regulatory subunit of phosphatidylinositol 3-kinase and prostate cancer risk: a prospective study**, Cancer Epidemiology, Biomarkers & Prevention, 2003, **12**:172-173.

[44] Yang Y, Hou H, Haller E, Nicosia S, and Bai W: **Suppression of FOXO1 activity by FHL2 through SIRT1-mediated deacetylation**, The EMBO Journal, .2005, **24(5)**:1021-1032.

[45] Kumar-Sinha C, Scott A, and Chinnaiyan A: **Recurrent gene fusions in prostate cancer**, Nature Reviews Cancer , 2009, **8**:497-511.

[46] Burke P, Gregg J, Bakhtiar B, Beckett L, Denardo G, Albrecht H, White RD, and De-Nardo S: **Characterization of MUC1 glycoprotein on prostate cancer for selection of targeting molecules**, International journal of oncology, 2006, **29(1)**:49-55.

[47] Moro L, Arbini A, Marra E, and Greco M: **Down-regulation of BRCA2 Expression by Collagen Type I Promotes Prostate Cancer Cell Proliferation**, The Journal of Biological Chemistry, 2005, **280(23)**:22482–22491.

[48] Shapiro E, Huang H, Ruoff R, Lee P, Tanese N, and Logan SK: **The heterochromatin protein 1 family is regulated in prostate development and cancer**, Journal of Urology, 2008, **179**:2435-2439.

[49] Mayeur GL, Kung W, Martinez A, Izumiya C, Chen DJ, and Kung H: **Ku is a novel transcriptional recycling coactivator of the androgen receptor in prostate cancer cells**, The Journal of Biological Chemistry, 2005, **280**:10827-10833.

[50] Okumura K, Mendoza M, Bachoo RM, DePinho RA, Cavenee WK, and Furnari FB: **PCAF Modulates PTEN Activity**, The Journal of Biological Chemistry, 2006, **281(36)**:26562-26568.

[51] Rosen E, Fan S, Pestell R, and Goldberg I: **BRCA1 in hormone-responsive cancers, Trends Endocrinol Metab**, 2003, **14(8)**:378–385.

[52] Rhodes D, Yu J, Shanker K, Deshpande N, Varambally R, Ghosh D, Barrette T, Pandey A, and Chinnaiyan A: **ONCOMINE: a cancer microarray database and integrated data-mining platform**, eoplasia, 2004, **6**:1-6.

[53] Halkidou K, Gaughan L, Cook S, Leung HY, Neal D, and Robson C: **Up-regulation and nuclear recruitment of HDAC1 in hormone refractory prostate cancer**, Prostate, 2004, **59**:177-189

[54] Chen Q, Watson JT, Marengo SR, Decker KS, Coleman I, Nelson PS, and Sikes RA: **Gene expression in the LNCaP human prostate cancer progression model: Progression associated expression in vitro corresponds to expression changes associated with prostate cancer progression in vivo**, Cancer Letters, 2006, **244**:274-288.

[55] Lu S, Lee J, Revelo M, Wang X, Lu S, and Dong Z: **Smad3 Is Overexpressed in Advanced Human Prostate Cancer and Necessary for Progressive Growth of Prostate Cancer Cells in Nude Mice**, Clinical Cancer Research, 2007, **13(19)**:5692-702.

[56] Yu C, Jin T, Chen W, Zhang P, Liu W, Guan H, Zhang J, Liu Q, and Jiang A: **Identification of Sp1-elements in the promoter region of human homeobox gene NKX3.1**, Molecular Biology Reports, 2009, **36(8)**:2353-2360.

[57] Drobnjak M, Osman I, and Scher H: **Overexpression of cyclin D1 is associated with metastatic prostate cancer to bone**, Clinical Cancer Research, 2000, **6**:1891-1895.

[58] Taneja S, Ha S, and Swenson NK: **ART-27, an androgen receptor coactivator regulated in prostate development and cancer**, The Journal of Biological Chemistry, 2004, **279**:13944–13952.

[59] True L, Coleman I, Hawley S, Huang C, Gifford D, Coleman R, Beer TM, Gelmann E, Datta M, Mostaghel E, Knudsen B, Vessella PLR, Lin D, Hood L, and Nelson PS: **A molecular correlate to the Gleason grading system for prostate adenocarcinoma**, Proceedings of the National Academy of Sciences USA, 2006, **103(29)**:10991–10996.

[60] Zhang L, Johnson M, Le K, Sato M, Ilagan R, Iyer M, Gambhir S, Wu L, and Carey M: **Interrogating androgen receptor function in recurrent prostate cancer**, Cancer Research, 2003, **63(15)**:4552-4560.

[61] Tu L, Yan X, Hood L, and Lin B: **Roteomics analysis of the interactome of N-myc downstream regulated gene 1 and its interactions with the androgen response program in prostate cancer cells**, Molecular & Cellular Proteomics, 2007, **6(4)**:575-588.

[62] Shoularsa K, Rodrigueza M, Thompsona T, and Markaverich BM: **Regulation of cell cycle and RNA transcription genes identified by microarray analysis of PC-3 human prostate cancer cells treated with luteolin**, The Journal of Steroid Biochemistry and Molecular Biology, 2010, **118(1-2)**:41-50.

[63] Lee DK, Duan HO, and Chang C: **From Androgen Receptor to the General Transcription Factor TFIIH**, The journal of biological chemistry, 2000, **275(13)**: 9308-9313.

[64] Khanim FL, Gommersall LM, Wood VH, Smith KL, Montalvo L, O'Neill LP, Xu Y, Peehl DM, Stewart PM, Turner BM, and Campbell MJ: **Altered SMRT levels disrupt vitamin D3 receptor signalling in prostate cancer cells**, Oncogene, 2004, **23(40)**:6712-6725.

[65] Anazawa Y, Nakagawa H, Furihara M, Ashida S, Tamura K, Yoshioka H, Shuin T, Fujioka T, Katagiri T, and Nakamura Y: **PCOTH, a novel gene overexpressed in prostate cancers, promotes prostate cancer cell growth through phosphorylation of oncoprotein TAF-Ibeta/SET**, Cancer Research, 2005, **65(11**):4578-4586.

[66] Berwick M, Satagopan JM, Ben-Porat L, Carlson A, Mah K, Henry R, Diotti R, Milton K, Pujara K, Landers T, Batish SD, Morales J, Schindler D, Hanenberg H, Hromas R, Levran O, and Auerbach AD: **Genetic heterogeneity among Fanconi anemia heterozygotes and risk of cancer**, Cancer Research, 2007, **67(19)**:9591-9596.

[67] Easton D: **Cancer risks in BRCA2 mutation carriers: The Breast Cancer Linkage Consortium**, Journal of the National Cancer Institute, 1999, **91(15)**:1310-1316.

[68] Singh AP, Bafna S, Chaudhary K, Venkatraman G, Smith L, Eudy JD, Johansson SL, Lin MF, and Batra SK: **Genome-wide expression profiling reveals transcriptomic variation and perturbed gene networks in androgen-dependent and androgen-independent prostate cancer cells**, Cancer Letter, 2008, **259(1)**:28-38.

[69] Tilki D, Oliveira-Ferrer L, Seitz M, Friedrich M, Stief C, and Ergun S: **Tumor angiogenesis regulation in prostate cancer by ceacam1**, The Journal of Urology, **179(4)**:419-419.

[70] Inokuchi J, Narula N, Yee DS, Skarecky DW, Lau A, Ornstein DK, and Tyson DR: **Annexin A2 positively contributes to the malignant phenotype and secretion of IL-6 in DU145 prostate cancer cells**, International Journal of Cancer, 2009, **124(1)**:68-74.

[71] Thorsen K, Sorensen KD, Brems-Eskildsen AS, Modin C, Gaustadnes M,. Hein AM, Kruhoffer M, Laurberg S, Borre M, Wang K, Brunak S, Krainer AR, Torring N, Dyrskjot L, Andersen CL, and Orntoft TF: **Alternative splicing in colon, bladder, and prostate cancer identified by exon array analysis**, Molecular & Cellular Proteomics, 2008, **7(7)**:214-224.

[72] Mohi MG, and Neel BG: **The role of SHP2 (PTPN11) in cancer**, Current Opinion in Genetics & Development, 2007, **17**: 23-30.

[73] Maher CA, Kumar-Sinha C, Cao X, Kalyana-Sundaram S, Han B, Jing X, Sam L, Barrette T, Palanisamy N, and Chinnaiyan AM: **Transcriptome sequencing to detect gene fusions in cancer**, Nature, 2009, **458(7234)**:97-101.
[74] Cifuentes E, Mataraza J, Yoshida A, Menon M, Sacks B, Barrack R, and Reddy G: **Physical and functional interaction of androgen receptor with calmodulin in prostate cancer cells**, Proceedings of the National Academy of Sciences USA, 2004, **101(2)**:464-469.
[75] Andrew S, Michael K, James E, Zohar D, Fernando B, Jr H, and Peter S: Defining Biochemical Recurrence of Prostate Cancer After Radical Prostatectomy: **A Proposal for a Standardized Definition, Journal of Clinical Oncology**, 2006, **24(24)**:3973-3978.
[76] Dunn GP, Sheehan KC, Old LJ, and Schreiber RD: **IFN Unresponsiveness in LNCaP Cells Due to the Lack of JAK1 Gene Expression, Cancer Research**, 2005, **65(8)**: 3447-3453.
[77] Fernando D, Lee S, Lou W, Xiao X, Beth P, Joel B, and Allen C: **Stat3 Enhances the Growth of LNCaP Human Prostate Cancer Cells in Intact and Castrated Male Nude Mice**, The Prostate, 2002, **52**:123-129.
[78] Bo H, Rohit M, Saravana M, Jindan Y, Anjana M, Robert J, Wang X, Gong Y, Wang L, Sunita S, Bharathi L, Rajal B, Sooryanarayana V, Nallasivam P, Scott A, Chandan K, and Arul M: **Transformation–Specific Aberrations: Identification of DDX5-ETV4 Fusion Protein in Prostate Cancer**, Cancer Research, 2008, **68**:7629–7637.
[79] Trevor J, Mira K, Lorena B, Allen D, Martin K, Dallas T, Karen N, Yang C, Alexander A, Michael M, Jim W, Peggy L, Marco M, and Richard A: **Sequence Variant Discovery in DNA Repair Genes from Radiosensitive and Radiotolerant Prostate Brachytherapy Patients**, Clinical Cancer Research, 2009, **15(15)**:5008-5016.
[80] Marika J, Kati P, Zhikang K, Kimmo J, Olli A, Teuvo T, Robert L, Jorma P, and Tapio V: **Expression of Androgen Receptor Coregulators in Prostate Cancer**, Clinical Cancer Research, 2004, **10**:1032-1040.
[81] Toshitaka S, Hideaki S, Noritaka M, Fuminori S, Yoshio N, Hiromitsu M, and Hidekatsu Y: **Sp1 and Sp3 transcription factors upregulate the proximal promoter of the human prostate-specific antigen gene in prostate cancer cells**, Archives of Biochemistry and Biophysics , 2005, **435**:291–302.
[82] Ravi V, Michael S, and Joseph D: **A Coregulatory Role for the Mediator Complex in Prostate Cancer Cell Proliferation and Gene Expression**, Cancer Research, 2007, **67(9)**:4034.

[83] Krajewska M, Turner B, Shabaik A, Krajewski S, and Reed JC: **Expression of BAG-1 protein correlates with aggressive behavior of prostate cancers**, Prostate, 2006, **66**:801-810.
[84] Lalande M and Calciano MA: **Molecular epigenetics of Angelman syndrome**, Cellular and Molecular Life Sciences, 2007, **64**:7-8.
